# Invited commentary: lubricating the rusty wheel, new insights into iron oxidizing bacteria through comparative genomics

**DOI:** 10.3389/fmicb.2014.00386

**Published:** 2014-07-30

**Authors:** Eric Altermann

**Affiliations:** ^1^Animal Nutrition and Health Group, AgResearch Ltd.Palmerston North, New Zealand; ^2^Centre of Research Excellence, Riddet Institute, Massey UniversityPalmerston North, New Zealand

**Keywords:** iron oxidizing bacteria, comparative genomics, taxonomy, life style adaptation, biogeochemistry, iron cycle

Iron is the fourth most abundant mineral in the Earth's lithosphere (Weber et al., 2006; Emerson et al., 2013), where it is present at a mean concentration of 5% (Hedrich et al., 2011). Iron can exist in two oxidation stages as ferrous (Fe(II)) and ferric (Fe(III)) iron and some bacteria and archaea have evolved to use iron as an obligate or facultative energy source, giving them the name “Iron Oxidizing Bacteria” (FeOB) and “Iron Oxidizing Archaea” (FeOA), respectively (Figure 1). The oxidation from Fe(II) to Fe(III) can occur under both oxic and anoxic conditions within a pH range between 0.5 to 8.4 (Edwards et al., 2000; Weber et al., 2006; Hedrich et al., 2011). Here, the microbially mediated oxidation of iron under (micro-)aerobic circum-neutral conditions will be discussed.

**Figure 1:**
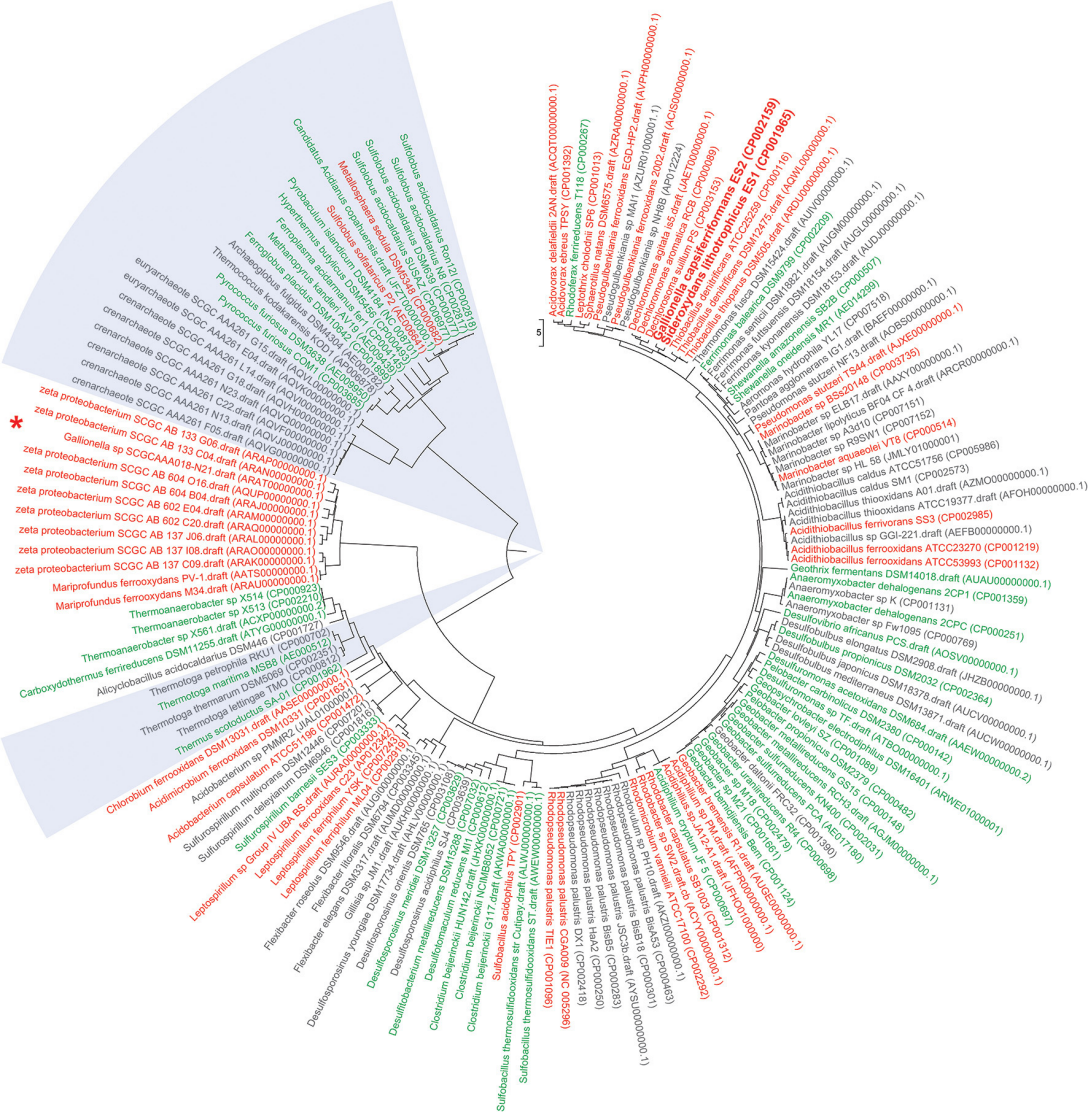
**Functional Genome Distribution Tree of iron oxidizing and iron reducing microbes**. 158 genomes of publicly available known (and predicted) iron oxidizing and iron reducing bacteria and archaea were included into a Functional Genome Distribution (FGD) analysis (Altermann, 2012). Briefly, draft genomes were downloaded in FASTA format, concatenated and their respective gene models predicted through an updated version of the GAMOLA annotation pipeline (Altermann and Klaenhammer, 2003). Complete genomes were downloaded in Genbank format. All 158 genomes were then subjected to an FGD analysis using the compACTor software. Briefly, within an FGD analysis complete ORFeomes are compared to each other, providing a snapshot of genome-to-genome similarities, rather than an ancestral outlook. In summary, the 158 genomes comprised 501,624 open reading frames (ORFs) and a total of 79,881,099 individual BLAST analyses were carried out to calculate the level of similarities between all genomes. The resulting dissimilarity matrix was then visualized in Mega6 (Tamura et al., 2013) using the UPGMA algorithm to infer a dissimilarity tree. The early draft sequence of *Leptothrix ochracea* L12 was found to be too incomplete and was removed from the final tree. Iron oxidizing bacteria and archaea are shown in red; iron reducing bacteria (FeRB) and archaea (FeRA) are depicted in green; likely iron oxidizing/reducing candidate microbes are indicated in gray. Archaeal iron oxidizers and reducers are highlighted in light blue sections. Accession numbers for respective Genbank or assembly entries are listed in brackets behind strain designations. *Sideroxydans lithotrophicus* ES1 and *Gallionella capsiferriformans* ES2 are shown in bold. Interestingly, *Sideroxydans lithotrophicus* ES1 and *Gallionella capsiferriformans* ES2 form their own sub-cluster and are most similar to *Thiobacillus* and *Dechloromonas* genomes. The recently published draft genome of *Gallionella* sp SCGCAAA018-N21 (marked by “^*^”) does not fall into the ES1/ES2 cluster, but groups within the larger zeta proteobacterium cluster. This may indicate the need to further investigate whether N21 represents a true *Gallionella* strain. In most instances, iron oxidizing and iron reducing microbes form their own separate cluster. There are notable exceptions (e.g., *Rhodoferax ferrireducens* T118 or *Geobacter bremensis* R1) and it may be possible that those microbes are able to carry out both iron oxidation and iron reduction, depending on their respective environmental circumstances. Most candidate iron oxidizing or iron reducing bacteria and archaea fall within distinct clusters that harbor examples of confirmed phenotypes. This may assist future research in systematically test for iron oxidizing or iron reducing phenotypes.

The biogeochemistry of iron poses several challenges to iron oxidizing microbes (for a detailed review see Weber et al., 2006). In circum-neutral environments and in the presence of oxygen, rapid abiotic oxidation of Fe(II) occurs, with half-life times of less than 60 s (Emerson et al., 2010). This abiotic oxidation to poorly soluble iron-oxyhydroxides reduces the availability of Fe(II) for biological process significantly. Microbial oxidation of Fe(II) therefore generally occurs in microaerophilic environments, which extend the half-life time of ferrous iron while at the same time increasing the amount of soluble Fe(II) (Bonnefoy and Holmes, 2012). Further, the oxidation from ferrous to ferric iron under low oxygen partial pressure increases the Gibbs free energy yield from 29 to −90 kJ mol^−1^ Fe(II) (Emerson et al., 2010).

Iron oxidizing bacteria and archaea have contributed on a global scale to shape the lithosphere (Emerson and Moyer, 2002; Weber et al., 2006). Evidence of their impact on the global iron cycle has been dated back to Pre-Cambrian age where they are thought to have potentially aided in the deposition of banded iron formations (Hedrich et al., 2011). Even today they have a major impact on terrestrial and aquatic systems. FeOB and FeOA can be found in habitats ranging from deep sea vents to freshwater systems and the rhizosphere and can form thick mats and deposits from a few millimeters to tens of centimeters thick. More recently, these microbes have made their appearance in man-made environments and industrial processes (Valdes et al., 2008). Most commonly known may be their contribution to biofouling and corrosion where the build-up of ferric iron can lead to a reduction in the water flow rates and water quality (Hedrich et al., 2011; Mcbeth et al., 2011).

Iron oxidizing microbes and their geological significance were first described in the 19th century (Ehrenberg, 1837; Harder, 1919). They are of key importance to global biogeochemical processes and more recently have gained interest for their impact on man-made environments (Hedrich et al., 2011). Yet, progress in understanding the ecology, physiology and genetic analysis of iron oxidizing microbes has been slow (Weber et al., 2006). Although iron oxidizing microbes can be readily observed in nature due to their striking morphology (such as the iconic stalk-forming *Gallionella*) and ability to form large microbial mats, they have proven elusive to cultivation under laboratory conditions. This elusiveness has caused confusion in the past, where taxonomy was inferred exclusively by morphological and ecological characteristics (Emerson et al., 2010). Recent advances in culturing techniques (Fabisch et al., 2013; Tischler et al., 2013) and culture-independent high-throughput DNA sequencing methods are providing a clearer phylogenetic picture of these bacteria (Emerson et al., 2010).

The recent availability of metagenomic data in particular has greatly accelerated our understanding of iron oxidizing microbes (Wang et al., 2011; Yelton et al., 2013). Only 54 bacterial and 13 archaeal genomes of FeOB are publicly accessible to date (Weber et al., 2006; Cardenas et al., 2010; Emerson et al., 2010; Hedrich et al., 2011), on which the emerging sequence-based taxonomic framework is built upon (Figure 1). New information from cultivated strains still refines this framework and the recently introduced class of marine Zetaproteobacteria (Emerson et al., 2007) is an excellent example of this rapid taxonomic evolution. An overview of publicly available genomes of iron oxidizing (and reducing) microbes and their relation to each other is presented in Figure 1.

While metagenomic datasets are valuable in improving our understanding of the presence and composition of iron oxidizing microbial communities (Ionescu et al., 2012; Kato et al., 2013; Singer et al., 2013), they rely on a robust gene-based taxonomic framework. Such a framework and detailed metabolic reconstruction of individual microbial strains require access to (draft) genome sequences and, consequently, to pure strains of iron oxidizing microbes (Kato et al., 2013). Despite the requirement for new strains of iron oxidizing microbes, isolation and characterization is challenging and time consuming. For example, *Gallionella* were first described in 1837 (Ehrenberg, 1837) and are amenable to cultivation. However, it was not until the mid-1990s where cultivation of two further strains, *Gallionella ferruginea and Gallionella capsiferriformans* ES-2, was successful (Hallbeck et al., 1993; Emerson and Moyer, 1997). Similarly, the isolation and characterization of other freshwater Fe(II) oxidizing Betaproteobacteria such as *Sideroxydans* sp. ES-1, that is metabolically flexible and can utilize reduced sulfur compounds and fix nitrogen, were also achieved only in the 1990s (Emerson and Moyer, 1997).

Since the initial description of both *Gallionella capsiferriformans* ES-2 and *Sideroxydans lithotrophicus* ES-1 a number of publications have investigated their morphological, phenotypical and industrial characteristics (Hallbeck and Pedersen, 1995; De Vet et al., 2012; Krepski et al., 2012), but no information was available on their genetic blueprint. The recent publication by Emerson et al. (2013), presenting the comparative analysis of genomic sequences of both *Sideroxydans lithotrophicus* ES-1 and *Gallionella capsiferriformans* ES-2, has filled this gap. By combining morphological characteristics with *in silico* comparative genomic analyses the authors were able to demonstrate that, although isolated from the same habitat, an iron-mat from ferruginous groundwater in Michigan (Emerson and Moyer, 1997), both strains belong to different genera in the recently proposed order Gallionellales. Importantly, the genomic comparison and metabolic pathway reconstruction highlighted a number of similarities and differences between both strains: 60% of the predicted genes were homologous at the 30% similarity level and both strains share a number of core metabolic capabilities for environmental sensing (chemotaxis), motility (pili), the uptake of nutrients through ABC transporters, energy metabolism, and detoxification of heavy metals, although strain ES-2 harbors a greater array of detoxification systems than ES-1 implying a superior ability to persist in habitats rich in environmental toxins. Notably, ~10% of both genomes is dedicated to environmental sensing and signal transduction which is a significantly higher number of genes than found in most other microbial genomes. Both strains harbor complete sets of chemotaxis genes and in combination with the elaborate environmental sensing array, controlled movement along the opposing gradients of Fe(II) and oxygen may facilitate optimal positioning of individual cells.

One of the problems aerobic Fe(II) oxidizing microbes must overcome is the presence of highly reactive oxygen species (ROS) that are produced via Fenton chemistry in the presence of oxygen and iron. Catalase and superoxide dismutase are the most common defense mechanisms against ROS and surprisingly both strains feature only a minimal set of catalase and peroxidase genes. This may be compensated by a number of truncated hemoglobins that are highly conserved and found in other FeOB such as *M. ferrooxydans* PV-1. These hemoglobins may represent an intracellular oxygen buffer that reduces the occurrence of ROS by binding oxygen under microaerophilic conditions and high concentrations of Fe(II).

Interestingly, strains ES-1 and ES-2 differ in their strategies to generate ATP. While ES-2 relies on the alternative complex III and a bacterial ATPase, ES-1 employs Cytochrome bc1, and a bacterial/archaeal-type ATPase in addition to the bacterial ATPase. The advantages of a second ATPase are presently unknown and the distribution of such redundant systems throughout the genomes of iron oxidizing microbes may provide clues to their respective niche adaptation in the future.

Both strains harbor prophage that may have been acquired independently and do not share homologous genes. However, the ES-1 prophage shares significant nucleic acid similarity (24%) to a prophage gene cluster identified in *Mariprofundus ferrooxydans PV-1* (Singer et al., 2011), possibly indicating shared hosts in the past. Neither of the two strains features CRISPR sequences that have long been associated with phage defense, posing an interesting question on the evolutionary pressure of bacteriophage in iron oxidizing communities. It is generally accepted that phage and their respective hosts are locked into an ongoing arms race that shapes their evolution (Stern and Sorek, 2011). Emerging genomes of iron oxidizing microbes will provide deeper insights into the defense mechanisms these microbes deploy to evade phage infection and killing.

Neither of the two strains features any of the unique extracellular structures such as sheaths or stalks often associated with FeOB. Genome analysis revealed that strain ES-1 feature a single large gene cluster dedicated to exopolymer synthesis, while ES-2 harbors two separate large EPS clusters in addition to two gene clusters possibly involved in cellulose synthesis. The presence of exopolysaccharide (EPS) gene clusters may provide clues how those strains prevent self-encrustation by iron-oxyhydroxides but further *in vitro* research is required to unravel these survival strategies.

Recently, the draft genome sequences of *Gallionella* sp. SCGC AAA018-N21 have been released (BioSample: SAMN02256456) and a future comparative genomics analyses will undoubtedly reveal further insights in lifestyle adaptation and contribute to a more robust taxonomic framework.

The research presented by Emerson et al. contributes significantly to our understanding of neutrophilic freshwater iron-oxidizing bacteria and their taxonomic relations to each other. However, the intriguing features found in both genomes—either commonly shared or specific to the individual strains—have created a large, new research space waiting to be investigated.

## Conflict of interest statement

The author declares that the research was conducted in the absence of any commercial or financial relationships that could be construed as a potential conflict of interest.

## References

[B1] Altermann E. (2012). Tracing lifestyle adaptation in prokaryotic genomes. Front. Microbiol. 3:48 10.3389/fmicb.2012.00048 22363326 PMC3282942

[B2] Altermann E. Klaenhammer T. R. (2003). GAMOLA: a new local solution for sequence annotation and analyzing draft and finished prokaryotic genomes. OMICS 7, 161–169 10.1089/153623103322246557 14506845

[B3] Bonnefoy V. Holmes D. S. (2012). Genomic insights into microbial iron oxidation and iron uptake strategies in extremely acidic environments. Environ. Microbiol. 14, 1597–1611 10.1111/j.1462-2920.2011.02626.x 22050575

[B4] Cardenas J. P. Valdes J. Quatrini R. Duarte F. Holmes D. S. (2010). Lessons from the genomes of extremely acidophilic bacteria and archaea with special emphasis on bioleaching microorganisms. Appl. Microbiol. Biotechnol. 88, 605–620 10.1007/s00253-010-2795-9 20697707

[B5] De Vet W. W. J. M. Dinkla I. J. T. Abbas B. A. Rietveld L. C. Van Loosdrecht M. C. M. (2012). *Gallionella spp.* in trickling filtration of subsurface aerated and natural groundwater. Biotechnol. Bioeng. 109, 904–912 10.1002/bit.24378 22105778

[B6] Edwards K. J. Bond P. L. Gihring T. M. Banfield J. F. (2000). An archaeal iron-oxidizing extreme acidophile important in acid mine drainage. Science 287, 1796–1799 10.1126/science.287.5459.1796 10710303

[B7] Ehrenberg C. G. (1837). Remarks on the real occurrence of fossil infusoria, and their extenisve diffusion. Taylor's Sci. Memoirs 1, 400–413, plate V.

[B8] Emerson D. Field E. K. Chertkov O. Davenport K. W. Goodwin L. Munk C. (2013). Comparative genomics of freshwater Fe-oxidizing bacteria: implications for physiology, ecology, and systematics. Front. Microbiol. 4:254 10.3389/fmicb.2013.00254 24062729 PMC3770913

[B9] Emerson D. Fleming E. J. Mcbeth J. M. (2010). Iron-oxidizing bacteria: an environmental and genomic perspective. Annu. Rev. Microbiol. 64, 561–583 10.1146/annurev.micro.112408.134208 20565252

[B10] Emerson D. Moyer C. (1997). Isolation and characterization of novel iron-oxidizing bacteria that grow at circumneutral pH. Appl. Environ. Microbiol. 63, 4784–4792 9406396 10.1128/aem.63.12.4784-4792.1997 PMC168801

[B11] Emerson D. Moyer C. L. (2002). Neutrophilic Fe-oxidizing bacteria are abundant at the Loihi Seamount hydrothermal vents and play a major role in Fe oxide deposition. Appl. Environ. Microbiol. 68, 3085–3093 10.1128/AEM.68.6.3085-3093.2002 12039770 PMC123976

[B12] Emerson D. Rentz J. A. Lilburn T. G. Davis R. E. Aldrich H. Chan C. (2007). A novel lineage of proteobacteria involved in formation of marine Fe-oxidizing microbial mat communities. PLoS ONE 2:e667 10.1371/journal.pone.0000667 17668050 PMC1930151

[B13] Fabisch M. Beulig F. Akob D. M. Kusel K. (2013). Surprising abundance of Gallionella-related iron oxidizers in creek sediments at pH 4.4 or at high heavy metal concentrations. Front. Microbiol. 4:390 10.3389/fmicb.2013.00390 PMC3866512 24385973

[B14] Hallbeck L. Pedersen K. (1995). Benefits associated with the stalk of Gallionella ferruginea, evaluated by comparison of a stalk-forming and a non-stalk-forming strain and biofilm studies *in situ*. Microb. Ecol. 30, 257–268 10.1007/BF00171933 24185563

[B15] Hallbeck L. Stahl F. Pedersen K. (1993). Phylogeny and phenotypic characterization of the stalk-forming and iron-oxidizing bacterium Gallionella ferruginea. J. Gen. Microbiol. 139, 1531–1535 10.1099/00221287-139-7-1531 8371116

[B16] Harder E. C. (1919). Iron-depositing bacteria and their geological relations, in US Geological Survey Professional Paper (Washington, DC), 85.

[B17] Hedrich S. Schlomann M. Johnson D. B. (2011). The iron-oxidizing proteobacteria. Microbiology 157, 1551–1564 10.1099/mic.0.045344-0 21511765

[B18] Ionescu D. Siebert C. Polerecky L. Munwes Y. Y. Lott C. Hausler S. (2012). Microbial and chemical characterization of underwater fresh water springs in the Dead Sea. PLoS ONE 7:e38319 10.1371/journal.pone.0038319 22679498 PMC3367964

[B19] Kato S. Chan C. Itoh T. Ohkuma M. (2013). Functional gene analysis of freshwater iron-rich flocs at circumneutral pH and isolation of a stalk-forming microaerophilic iron-oxidizing bacterium. Appl. Environ. Microbiol. 79, 5283–5290 10.1128/AEM.03840-12 23811518 PMC3753938

[B20] Krepski S. T. Hanson T. E. Chan C. S. (2012). Isolation and characterization of a novel biomineral stalk-forming iron-oxidizing bacterium from a circumneutral groundwater seep. Environ. Microbiol. 14, 1671–1680 10.1111/j.1462-2920.2011.02652.x 22151253

[B21] Mcbeth J. M. Little B. J. Ray R. I. Farrar K. M. Emerson D. (2011). Neutrophilic iron-oxidizing “zetaproteobacteria” and mild steel corrosion in nearshore marine environments. Appl. Environ. Microbiol. 77, 1405–1412 10.1128/AEM.02095-10 21131509 PMC3067224

[B22] Singer E. Emerson D. Webb E. A. Barco R. A. Kuenen J. G. Nelson W. C. (2011). Mariprofundus ferrooxydans PV-1 the first genome of a marine Fe(II) oxidizing Zetaproteobacterium. PLoS ONE 6:e25386 10.1371/journal.pone.0025386 21966516 PMC3179512

[B23] Singer E. Heidelberg J. F. Dhillon A. Edwards K. J. (2013). Metagenomic insights into the dominant Fe(II) oxidizing Zetaproteobacteria from an iron mat at Lo ihi, Hawai l. Front. Microbiol. 4:52 10.3389/fmicb.2013.00052 23518919 PMC3603346

[B24] Stern A. Sorek R. (2011). The phage-host arms race: shaping the evolution of microbes. Bioessays 33, 43–51 10.1002/bies.201000071 20979102 PMC3274958

[B25] Tamura K. Stecher G. Peterson D. Filipski A. Kumar S. (2013). MEGA6: molecular evolutionary genetics analysis version 6.0. Mol. Biol. Evol. 30, 2725–2729 10.1093/molbev/mst197 24132122 PMC3840312

[B26] Tischler J. S. Jwair R. J. Gelhaar N. Drechsel A. Skirl A.-M. Wiacek C. (2013). New cultivation medium for “Ferrovum” and Gallionella-related strains. J. Microbiol. Methods 95, 138–144 10.1016/j.mimet.2013.07.027 23954479

[B27] Valdes J. Pedroso I. Quatrini R. Dodson R. J. Tettelin H. Blake R. (2008). Acidithiobacillus ferrooxidans metabolism: from genome sequence to industrial applications. BMC Genomics 9:597 10.1186/1471-2164-9-597 19077236 PMC2621215

[B28] Wang J. Vollrath S. Behrends T. Bodelier P. L. Muyzer G. Meima-Franke M. (2011). Distribution and diversity of Gallionella-like neutrophilic iron oxidizers in a tidal freshwater marsh. Appl. Environ. Microbiol. 77, 2337–2344 10.1128/AEM.02448-10 21317256 PMC3067411

[B29] Weber K. A. Achenbach L. A. Coates J. D. (2006). Microorganisms pumping iron: anaerobic microbial iron oxidation and reduction. Nat. Rev. Microbiol. 4, 752–764 10.1038/nrmicro1490 16980937

[B30] Yelton A. P. Comolli L. R. Justice N. B. Castelle C. Denef V. J. Thomas B. C. (2013). Comparative genomics in acid mine drainage biofilm communities reveals metabolic and structural differentiation of co-occurring archaea. BMC Genomics 14:485 10.1186/1471-2164-14-485 23865623 PMC3750248

